# Arginine promotes *Proteus mirabilis* motility and fitness by contributing to conservation of the proton gradient and proton motive force

**DOI:** 10.1002/mbo3.194

**Published:** 2014-08-07

**Authors:** Chelsie E Armbruster, Steven A Hodges, Sara N Smith, Christopher J Alteri, Harry L T Mobley

**Affiliations:** 1 Department of Microbiology and Immunology, University of Michigan Medical SchoolAnn Arbor, Michigan, 48104

**Keywords:** Arginine decarboxylase, *Proteus mirabilis*, proton motive force, swarming, swimming, UTI

## Abstract

Swarming contributes to *Proteus mirabilis* pathogenicity by facilitating access to the catheterized urinary tract. We previously demonstrated that 0.1–20 mmol/L arginine promotes swarming on normally nonpermissive media and that putrescine biosynthesis is required for arginine-induced swarming. We also previously determined that arginine-induced swarming is pH dependent, indicating that the external proton concentration is critical for arginine-dependent effects on swarming. In this study, we utilized survival at pH 5 and motility as surrogates for measuring changes in the proton gradient (ΔpH) and proton motive force (*μ*H^+^) in response to arginine. We determined that arginine primarily contributes to ΔpH (and therefore *μ*H^+^) through the action of arginine decarboxylase (*speA*), independent of the role of this enzyme in putrescine biosynthesis. In addition to being required for motility, *speA* also contributed to fitness during infection. In conclusion, consumption of intracellular protons via arginine decarboxylase is one mechanism used by *P. mirabilis* to conserve ΔpH and *μ*H^+^ for motility.

## Introduction

Urinary tract infection (UTI) is one of the most common infections worldwide and causes a significant healthcare burden, with ∼50% of women and 12% of men experiencing a UTI in their lifetime (Foxman and Brown 2003; Klevens et al. 2007; Foxman 2010). In addition to uncomplicated UTI, the majority of patients undergoing long-term catheterization will experience at least one episode of catheter-associated UTI (CaUTI), making it the most common hospital-acquired infection (Morris et al. 1999; Jacobsen et al. 2008; Hooton et al. 2010). If the UTI does not resolve, resulting complications can include the development of acute pyelonephritis and bacteremia.

The Gram-negative bacterium *Proteus mirabilis* is responsible for ∼3% of all nosocomial infections in the United States and up to 44% of CaUTIs (O’Hara et al. 2000; Nicolle 2005; Jacobsen et al. 2008). *Proteus mirabilis* possesses numerous virulence factors that contribute to the establishment of UTI (Coker et al. 2000; Jacobsen et al. 2008; Armbruster and Mobley 2012). In addition to typical virulence factors, *P. mirabilis* differentiates into elongated swarm cells capable of migrating across catheters to reach the bladder (Stickler and Hughes 1999; Sabbuba et al. 2002; Jacobsen et al. 2008).

Current research has revealed a complex regulatory network governing swarm cell differentiation, with most factors acting on the flagellar master regulator FlhD_2_C_2_ as described in recent reviews (Rather 2005; Morgenstein et al. 2010; Armbruster and Mobley 2012). However, little is known concerning how *P. mirabilis* coordinates swarm cell differentiation and migration. Swarming appears to be cell-density dependent, yet known quorum sensing systems are not involved (Belas et al. 1998; Schneider et al. 2002). The polyamine putrescine has been proposed as a signaling molecule for coordination of swarming in *P. mirabilis* as migration of the swarm requires putrescine synthesis via arginine decarboxylase (SpeA) and agmatinase (SpeB) (Sturgill and Rather 2004; Armbruster et al. 2013), as well as putrescine uptake via PlaP (Kurihara et al. 2013). Putrescine is also a component of the core region of *P. mirabilis* lipopolysaccharide (Vinogradov and Perry 2000) and may be important for changes in cell wall composition during differentiation.

In an endeavor to understand how *P. mirabilis* determines when an environment is permissive for swarming, we previously identified five cues present in normal human urine capable of inducing motility under normally nonpermissive conditions (Armbruster et al. 2013). One of the cues, l-arginine, contributes to the primary pathway for putrescine biosynthesis through a two-step reaction: arginine decarboxylase (*speA*) converts arginine to agmatine and agmatinase (*speB*) converts agmatine to putrescine (Pearson et al. 2008). Putrescine is critical for swarming, and must be produced through this pathway or exogenously supplied and imported in order for *P. mirabilis* to migrate across permissive media (Sturgill and Rather 2004; Armbruster et al. 2013; Kurihara et al. 2013). We previously showed that this pathway must also be intact or complemented by the addition of exogenous putrescine for swarming to occur under normally nonpermissive conditions in response to swarming cues such as arginine (Armbruster et al. 2013). However, the ability of arginine to promote swarming appears separate from the generation of putrescine as other components of this pathway, including agmatine and putrescine itself, were not capable of promoting swarming under the conditions tested (Armbruster et al. 2013). Thus, arginine may promote swarming through an additional mechanism independent of its role in putrescine biosynthesis.

*Proteus mirabilis* swarming is known to be intimately connected to energy metabolism (Armitage 1981; Alteri et al. 2012), which is not surprising considering that flagellar rotation is dependent on proton motive force (*μ*H^+^) (Gabel and Berg 2003). As swarm cells exhibit increased flagellum density and are capable of faster movement than vegetative cells (Tuson et al. 2013), a substantial *μ*H^+^ is likely required to fuel rotation of the hundreds to thousands of flagella expressed by *P. mirabilis* swarm cells. Metabolic activity is highest when *P. mirabilis* is preparing to swarm and surprisingly low during actual migration, indicating that swarm cells are almost entirely devoted to flagellar-mediated motility during swarm front migration (Armitage 1981; Pearson et al. 2010). The energy used to fuel swarming was previously thought to be generated through fermentation (Falkinham and Hoffman 1984), which is unusual as fermentation is not as energetically favorable as respiration. Under aerobic conditions, oxygen is used as the terminal electron acceptor and the greatest number of protons can be pumped across the cytoplasmic membrane through the respiratory chain, producing a strong *μ*H^+^. Under anaerobic conditions, an alternative electron acceptor can be used for the respiratory chain, but the process releases less energy. During fermentation, however, ATP must be consumed to pump protons and conserve the pH and charge gradients (ΔpH and Δ*ψ*), making it difficult to maintain *μ*H^+^ and fuel flagellar rotation.

To address this issue, recent work in our laboratory investigated the ability of *P. mirabilis* to use a complete oxidative tricarboxylic acid (TCA) cycle during swarming under aerobic conditions without apparently requiring oxygen as the terminal electron acceptor (Alteri et al. 2012). Based on this study, it was proposed that *P. mirabilis* maintains *μ*H^+^ and drives flagellar rotation through the oxidative TCA cycle while using an anaerobic respiratory chain in which fumarate metabolism plays a critical role (Alteri et al. 2012). However, it was still not clear how *P. mirabilis* could support the magnitude of *μ*H^+^ required to fuel swarming using an anaerobic respiratory chain.

Notably, arginine metabolism can influence both components of *μ*H^+^ (ΔpH and Δ*ψ*) through a mechanism that is used by some bacterial species to tolerate acidic conditions (Foster 2004). Arginine decarboxylase replaces the *α*-carboxyl group of arginine (+1 charge) with a proton recruited from the cytoplasm to generate agmatine (+2 charge), thus consuming an intracellular proton and therefore contributing to ΔpH (Gong et al. 2003; Foster 2004). Agmatine export can then be coupled to arginine import, influencing Δ*ψ*. Acid-tolerant bacteria utilize this and related systems to alter membrane potential and impede movement of external protons across the membrane, thereby allowing for survival in acidic environments. While *P. mirabilis* is not known to be acid-tolerant, it may utilize arginine decarboxylation to contribute to *μ*H^+^ in a similar manner.

In this study, we utilized mutants defective in arginine transport or putrescine biosynthesis to elucidate one mechanism by which arginine promotes motility and fitness. Using survival at pH 5 and motility as surrogates for measuring changes in the proton gradient (ΔpH) and proton motive force (*μ*H^+^), we determined that arginine contributes to conservation of *μ*H^+^ and that arginine decarboxylase (*speA*) contributes to *P. mirabilis* swimming and swarming motility independent of its role in putrescine biosynthesis. Loss of *speA* also resulted in a fitness defect that was not observed for a *speBF* double mutant lacking both routes for putrescine biosynthesis. We thus conclude that consumption of intracellular protons via arginine decarboxylation is one mechanism used by *P. mirabilis* to conserve ΔpH and *μ*H^+^ to support motility. This mechanism may also shed light on how *P. mirabilis* supports vigorous swarming while utilizing an anaerobic respiratory chain.

## Experimental Procedures

### Bacterial strains and culture conditions

Strains used in this study are listed in Table1. Bacteria were routinely cultured at 37°C with aeration. Swarming on permissive medium was assessed using swarm agar (10 g L^−1^ tryptone, 5 g L^−1^ yeast extract, 5 g L^−1^ NaCl solidified with 1.5% agar), which will be referred to as SWA, supplemented with various concentrations of l-arginine (Research Products International Corp., Mount Prospect, IL, USA). Media were supplemented with chloramphenicol (20 *μ*g mL^−1^), ampicillin (100 *μ*g mL^−1^), and kanamycin (25 *μ*g mL^−1^) as required.

**Table 1:** *Proteus mirabilis* strains and constructs used in this study.

Strain	Description	Reference
HI4320	*Proteus mirabilis* isolated from the urine of an elderly, long-term–catheterized woman	Mobley and Warren (1987)
*speA*	Kan^R^ insertion disrupting arginine decarboxylase	This study
*speB*	Kan^R^ insertion disrupting agmatinase	Armbruster et al. (2013)
*speBF*	Kan^R^ insertion disrupting ornithine decarboxylase was excised and an additional Kan^R^ was inserted into agmatinase	Armbruster et al. (2013)
*artM*	Kan^R^ insertion disrupting the arginine transporter permease subunit	This study
*ydgI*	Kan^R^ insertion disrupting an arginine:ornithine antiporter	This study

### Construction of mutants

TargeTron (Sigma, St Louis, MO, USA) was utilized to insert a kanamycin resistance cassette into genes of interest according to the manufacturer's instructions as described previously (Pearson and Mobley 2007). Primer sequences for intron reprogramming and PCR verification of mutants are provided in Table S1.

### Growth curves

Overnight cultures of bacteria were diluted 1:100 in Luria broth (LB, 10 g L-1 tryptone, 5 g L-1 yeast extract, 0.5 g L-1 NaCl). Where indicated, LB was supplemented with 10 or 20 mmol/L arginine, buffered with 10 mmol/L 4-(2-hydroxyethyl)-1-piperazineethanesulfonic acid (HEPES; Sigma), and adjusted to pH 5 or 7. A Bioscreen-C Automated Growth Curve Analysis System (Growth Curves, USA) was utilized to generate growth curves. Cultures were incubated at 37°C with continuous shaking, and OD_600_ readings were taken every 15 min for 17–24 h.

### Swarming motility

SWA plates (with or without arginine, agmatine, and putrescine) were inoculated with 5 *μ*L of an overnight culture. Plates were incubated at 37°C for 18 h, and swarm colony diameter was measured.

### Acid resistance experiments

Overnight cultures were diluted 1:50 into 5 mL LB and cultured at 37°C with aeration to log phase (2–3 h). Cultures were centrifuged (16,060*g*, 5 min, 25°C) and resuspended in 10 mmol/L 2-(*N*-morpholino)-ethanesulfonic acid (MES; Sigma) buffer to an OD_600_ of ∼1.0. The bacterial suspension was diluted 1:10 into 1 mL of 10 mmol/L MES adjusted to pH 7, 5, or 2.5 using HCl and KOH, with or without 20 mmol/L arginine. Carbonyl cyanide 3-chlorophenylhydrazone (CCCP; 100 *μ*mol/L) diluted in dimethyl sulfoxide (DMSO) was added as indicated to depolarize the inner membrane, and control cultures were incubated with DMSO alone. Samples were incubated at 37°C with aeration for 1 h and plated to determine CFU mL^−1^ using an Autoplate 4000 spiral plater (Spiral Biotech, Norwood, MA, USA). Colonies were enumerated using a QCount automated plate counter (Spiral Biotech).

### pH of overnight cultures

Overnight cultures of *P. mirabilis* HI4320 and *speA* were diluted 1:100 into 3 mL LB buffered with 10 mmol/L HEPES and adjusted to pH 5 with concentrated HCl or KOH after the addition of 10 or 20 mmol/L arginine. Cultures were incubated at 37°C with aeration. After 18 h, pH was determined to the nearest increment of 0.5 using pH test paper (Fisherbrand, Pittsburgh, PA, USA).

### Swimming motility

Motility agar plates (MOT, 10 g L^−1^ tryptone, 0.5 g L^−1^ NaCl, 3 g L^−1^ agar) with or without 20 mmol/L arginine were stab inoculated with an overnight culture of *P. mirabilis* or isogenic mutants. MOT plates were incubated at 30°C for 18 h prior to measurement of swimming diameter.

### Effect of arginine on *μ*H^+^ during swimming and swarming

Six-millimeter blank paper disks (BD) were impregnated with 10 *μ*L of 10 mmol/L CCCP or DMSO as a negative control and placed on SWA or MOT with or without 20 mmol/L arginine. SWA plates were inoculated with 5 *μ*L from an overnight culture and incubated at 37°C for 18 h, and MOT plates were stab inoculated from an overnight culture and incubated at 30°C for 18 h. The zone of inhibition of motility was measured by determining the distance (mm) from the swarm or swim edge to the outermost edge of the disk. For experiments using the *speA* mutant, 50 *μ*L of 50 mmol/L putrescine was spread on SWA plates to chemically complement swarming defects.

### Mouse model of ascending UTI

Co-challenge experiments with CBA/J mice were carried out as described previously (Johnson et al. 1987) using a modification of the Hagberg et al. (1983) protocol. Briefly, bacteria were cultured overnight in 5 mL LB, diluted to an OD_600_ of ∼0.2, mixed 1:1, and mice were inoculated transurethrally with 50 *μ*L of 2 × 10^8^ CFU mL^−1^ (1 × 10^7^ CFU per mouse). Mice were euthanized 7 days postinoculation and urine, bladder, and kidneys were harvested and transferred into sterile tubes containing 3 mL phosphate-buffered saline (0.128 mol/L NaCl, 0.0027 mol/L KCl, pH 7.4). Tissues were homogenized using an Omni TH homogenizer (Omni International Kennesaw, GA, USA) and plated onto LB agar using an Autoplate 4000 spiral plater (Spiral Biotech). Colonies were enumerated with a QCount automated plate counter (Spiral Biotech). A competitive index (CI) was calculated for each organ with a bacterial load greater than the limit of detection by determining the ratio of mutant to wild type as follows:
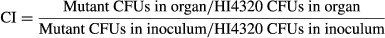


A CI of 1 indicates that the mutant colonized the organ to a similar level as *P. mirabilis* HI4320, a CI <1 indicates that the mutant was outcompeted by wild type, and a CI >1 indicates that wild type was outcompeted by the mutant.

### Statistics

Significance was determined by unpaired Student's *t*-test, two-way ANOVA, and the Wilcoxon signed rank test as indicated. All *P* values are two tailed at a 95% confidence interval. Analyses were performed using GraphPad Prism, version 6.04 (GraphPad Software, San Diego, CA).

## Results

### Arginine contributes to ΔpH and *μ*H^+^

We previously determined that arginine was most effective at promoting swarming when agar was mildly acidic and less effective when external pH increased (Armbruster et al. 2013). This observation suggests that swarming in response to arginine is related to proton distribution, and therefore possibly proton motive force (*μ*H^+^). To directly measure an effect on *μ*H^+^, we first attempted to use the carbocyanine dye 3,3′-diethyloxacarbocyanine iodide (DiOC_2_), which self-associates at high intracellular concentrations (Sims et al. 1974). However, *μ*H^+^ could not be measured with this technique as *P. mirabilis* HI4320 failed to take up DiOC_2_ under all conditions tested, even when outer membrane integrity was compromised by incubation with EDTA (data not shown).

**Figure 1:**
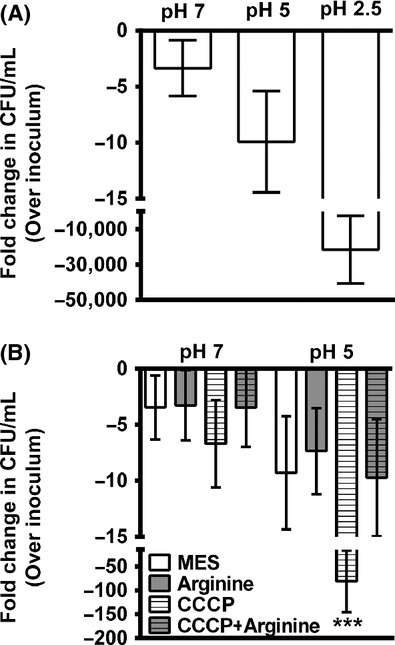
Arginine contributes to *μ*H^+^ in *Proteus mirabilis*. (A) *Proteus mirabilis* HI4320 was incubated for 60 min in 10 mmol/L MES buffer at pH 7, 5, or 2.5, and fold change in CFU mL^−1^ were determined. Error bars indicate mean ± SD for five independent experiments. (B) *Proteus mirabilis* HI4320 was incubated for 60 min in 10 mmol/L MES buffer at pH 7 or 5. Viable bacteria were enumerated for MES buffer with and without 10 mmol/L CCCP and 20 mmol/L arginine. CFU mL^−1^ were normalized to the inoculum. Error bars represent fold change over CFU mL^−1^ in the inoculum ± SD for at least three independent experiments. ***P <* *0.001 by Student's *t*-test compared to incubation in unsupplemented pH 5 MES.

As bacteria can modulate ΔpH and/or Δ*ψ* to promote survival during exposure to acidic conditions (Foster 2004), an alternate method for observing changes in the proton gradient (and therefore *μ*H^+^) involves tolerance of acidic conditions. To first determine whether *P. mirabilis* tolerates acidity, *P. mirabilis* HI4320 was incubated for 60 min in MES buffer adjusted to pH 7, 5, or 2.5 (Fig.1A). *Proteus mirabilis* HI4320 was not able to maintain viability during incubation at pH 2.5. However, incubation at pH 5 was tolerated with only a modest decrease in viability. We can therefore utilize survival in pH 5 MES buffer to investigate the contribution of arginine to conservation of the proton gradient and *μ*H^+^.

Viability at pH 5 was next tested in the presence of the protonophore chlorophenylhydrazone (CCCP), which depolarizes the electrochemical gradient across the inner membrane thereby collapsing membrane potential and *μ*H^+^ (Fig.1B). Importantly, neither CCCP nor arginine significantly impacted viability during incubation in pH 7 MES buffer. However, depolarization of the membrane in pH 5 MES buffer resulted in a dramatic decrease in viability, and this effect was abrogated in the presence of 20 mmol/L arginine. The data therefore indicate that *P. mirabilis* can utilize arginine to conserve membrane potential and *μ*H^+^.

### Arginine decarboxylase is required for arginine-dependent effects on ΔpH and *μ*H^+^

Our previous data indicated that arginine may promote swarming through an additional mechanism independent of its role in putrescine biosynthesis (Armbruster et al. 2013). Based on the ability of arginine to counteract the effects of CCCP, *P. mirabilis* is clearly capable of utilizing this amino acid to influence membrane potential, possibly through the action of arginine decarboxylase (*speA*). Arginine decarboxylase contributes to the distribution of protons across the membrane (ΔpH) as this reaction consumes a cytoplasmic proton during generation of agmatine (Gong et al. 2003; Foster 2004). Acid-tolerant bacteria can utilize this reaction and an arginine:agmatine antiporter as one mechanism for altering membrane potential (and therefore *μ*H^+^) to impede movement of external protons across the membrane, thereby allowing for survival in acidic environments. Even though *P. mirabilis* HI4320 was not acid tolerant (Fig.1), it may utilize arginine decarboxylation through a similar mechanism to contribute to *μ*H^+^ for survival in the presence of CCCP at pH 5.

**Figure 2:**
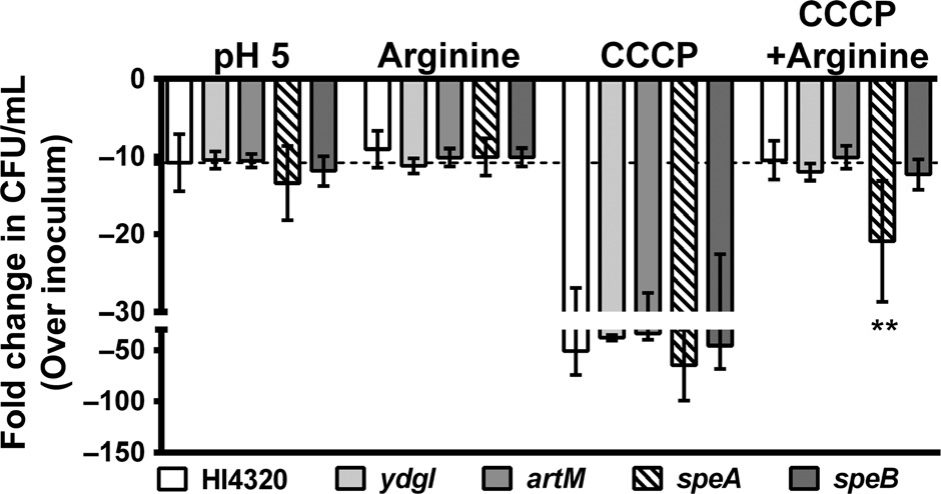
Arginine decarboxylase contributes to *μ*H^+^. *Proteus mirabilis* and isogenic mutants were incubated for 60 min in 10 mmol/L MES buffer at pH 5. Viable bacteria were enumerated for MES buffer with and without 10 mmol/L CCCP and 20 mmol/L arginine. CFU mL^−1^ were normalized to the inoculum. Dashed line indicates *P. mirabilis* HI4320 viability in MES buffer at pH 5 without supplement. Error bars represent fold change over CFU mL^−1^ in the inoculum ± SD for at least four independent experiments. **P <* *0.001 by Student's *t*-test compared to *P. mirabilis* CCCP plus arginine, *speA* CCCP, and *speA* arginine.

*Proteus mirabilis* HI4320 does not encode a homolog of the *Escherichia coli* or *Salmonella enterica* serovar Typhimurium arginine:agmatine antiporter (*adiC*), but it does encode an arginine:ornithine antiporter (*ydgI*) with 26% identity to *E. coli* AdiC by blastx, as well as the Art system for arginine import. To investigate genetic requirements for arginine-dependent survival at pH 5 in the presence of CCCP, insertion mutants of *speA, ydgI*, and *artM* were generated using TargeTron and tested for survival at pH 5 (Fig.2). All of the mutants tolerated incubation at pH 5 and the presence of arginine to a similar extent as *P. mirabilis* HI4320, and all exhibited a decrease in viability during incubation with CCCP. Importantly, arginine protected all mutants from the effects of CCCP except *speA*, which exhibited significantly reduced viability compared to *P. mirabilis* HI4320 and to incubation in pH 5 MES with arginine. Notably, the arginine transporter mutants *artM* and *ydgI* did not exhibit defects, suggesting that the presence of either transporter is sufficient for importing arginine and an antiport mechanism is not required under these conditions. These data therefore support a role for l-arginine and arginine decarboxylase in conservation of the proton gradient and *μ*H^+^. Attempts to generate an *artM/ydgI* double mutant were not successful (data not shown), so the role of arginine transport in conservation of *μ*H^+^ could not be confirmed.

To verify that the contribution of *speA* to proton distribution observed in these experiments with MES buffer are applicable to LB, *P. mirabilis* HI4320 and the *speA* mutant were cultured in lightly buffered LB broth adjusted to pH 5 after supplementation with 10 or 20 mmol/L arginine, and supernatant pH was measured following overnight incubation (Table2). We observed that *P. mirabilis* HI4320 raises the pH to ∼7 during overnight culture and supplementation with arginine promoted further alkalinization to pH ∼8.5, both of which were dependent on *speA*. Similar results were obtained when measuring growth in pH 5 LB broth, although it is important to note that the *speA* mutant did not exhibit a growth defect at pH 7 (Fig. S1). Thus, arginine and arginine decarboxylase contribute to proton distribution and alkalinization of LB.

**Table 2:** Culture pH following overnight incubation.

Strain	pH 5 LB	10 mmol/L arginine	20 mmol/L arginine	*P* value^1^
HI4320	7.35 ± 1.88	8.06 ± 1.61	8.69 ± 0.46	–
*speA*	5.19 ± 0.35	5.83 ± 1.60	6.87 ± 1.90	0.0001

Determined by two-way ANOVA.

### SpeA contributes to conservation of ΔpH and *μ*H^+^ during swimming

**Figure 3:**
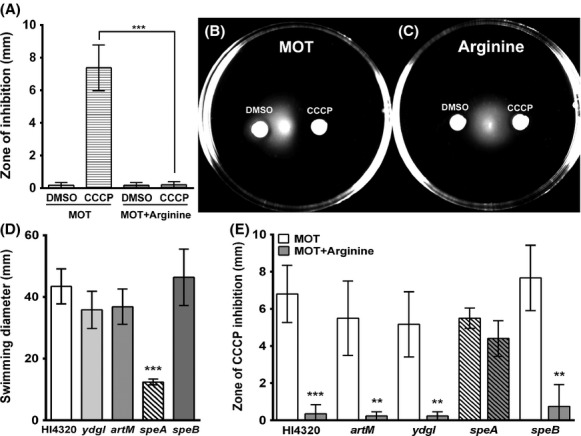
Arginine promotes swimming through conservation of *μ*H^+^. (A–C) Motility agar plates (MOT) with or without 20 mmol/L arginine were stab inoculated with *Proteus mirabilis* HI4320. Disks impregnated with DMSO or 10 mmol/L CCCP were placed on the surface of the plates, and the zone of inhibition of motility was measured after an 18 h incubation at 30°C. (A) Measurement of the zone of inhibition of swimming. Error bars represent mean ± SD for four independent experiments with three replicates each. ***P <* *0.001 by Student's *t*-test. (B) Representative image of *P. mirabilis* HI4320 swimming in MOT. (C) Representative image of *P. mirabilis* HI4320 swimming in MOT supplemented with arginine. (D) Swimming diameter was measured for *P. mirabilis* HI4320 and isogenic mutants after an 18-h incubation at 30°C, **P <* *0.01 and ***P <* *0.001 by Student's *t*-test compared to *P. mirabilis* HI4320. (E) Measurement of the zone of inhibition of swimming for *P. mirabilis* HI4320 and isogenic mutants in MOT agar with or without 20 mmol/L arginine. **P <* *0.01 and ***P <* *0.001 by Student's *t*-test compared to MOT.

Swimming motility requires *μ*H^+^ to fuel flagellar rotation (Gabel and Berg 2003), and this form of motility is inhibited when membrane potential is disrupted by CCCP. To demonstrate that arginine contributes to motility through conservation of *μ*H^+^, we next tested the ability of arginine to counter inhibition of swimming in motility agar (MOT) by CCCP (Fig.3). As expected, disks containing only DMSO had no effect on swimming, but CCCP inhibited motility as evidenced by development of a zone of inhibition extending ∼7 mm from the disk (Fig.3A and B). Importantly, swimming was not inhibited in plates containing arginine (Fig.3A and C). Thus, arginine counters the effect of CCCP and allows *P. mirabilis* to continue swimming.

As swimming requires *μ*H^+^, mutations that influence *μ*H^+^ should affect motility and interfere with the ability of arginine to rescue swimming in the presence of CCCP. Total swimming diameter was first measured for *P. mirabilis* HI4320 and isogenic mutants (Fig.3D). The only mutant that exhibited decreased swimming diameter was *speA*, while loss of *speB* did not affect swimming diameter, indicating that the role of *speA* in conservation of ΔpH and *μ*H^+^ can be separated from its role in putrescine biosynthesis. Supplementation with arginine failed to significantly alleviate the inhibitory effects of CCCP for the *speA* mutant while swimming was restored for all other mutants, including *speB* (Fig. 3E).

### SpeA contributes to conservation of ΔpH and *μ*H^+^ during swarming

**Figure 4:**
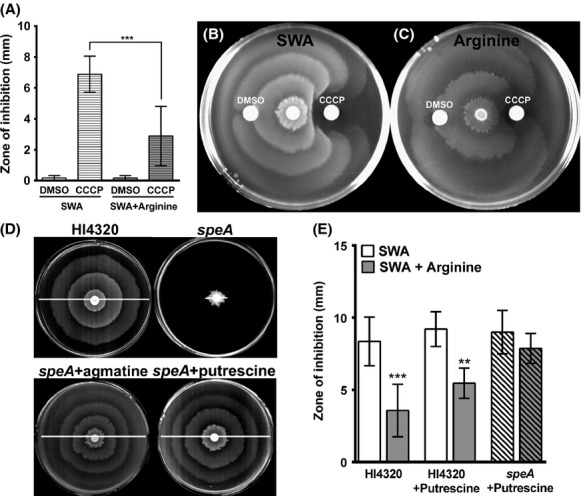
Arginine and arginine decarboxylase promote swarming through conservation of *μ*H^+^. (A–C) SWA with or without 20 mmol/L arginine were inoculated with *Proteus mirabilis* HI4320. Disks impregnated with DMSO or 10 mmol/L CCCP were placed on the surface of the plates and the zone of inhibition of motility was measured. (A) Measurement of the zone of inhibition of swarming. Error bars represent mean ± SD for four independent experiments with three replicates each. ***P <* *0.001 by Student's *t*-test. (B) Representative image of swarming on SWA. (C) Representative image of swarming on SWA supplemented with arginine. (D) Representative images of SWA plates inoculated with *P. mirabilis* HI4320 and isogenic mutants after incubation at 37°C for 18 h. The *speA* mutant was chemically complemented by 20 mmol/L agmatine or by spreading 50 *μ*L of 50 mmol/L putrescine on the surface of the plate. White lines indicate total swarm diameter. (E) Measurement of the zone of inhibition of swarming for *P. mirabilis* HI4320 and *speA*. Where indicated, 50 *μ*L of 50 mmol/L putrescine was spread on the surface of the plates. **P <* *0.01 and ***P <* *0.001 by Student's *t*-test.

Swarming also requires *μ*H^+^ but is more complex due to the requirement for differentiation to an elongated swarm cell and multicellular interactions. We therefore wanted to determine whether CCCP also inhibits swarming, and whether inhibition could be similarly alleviated by arginine. DMSO did not perturb swarming, but a zone of inhibition was visible around disks containing CCCP (Fig.4A and B). Similar to swimming motility, arginine significantly decreased inhibition of swarming by CCCP (Fig.4A and C), indicating that *P. mirabilis* can utilize arginine to promote swarming by conserving ΔpH and *μ*H^+^.

We next wanted to determine whether *speA* contributes to conservation of ΔpH and *μ*H^+^ during swarming. Consistent with previous reports (Sturgill and Rather 2004), the *speA* mutant exhibited a severe swarming defect that could be chemically complemented with putrescine (Fig.4D). Swarming was also complemented by agmatine, confirming that the *speA* mutation is not polar on *speB* (Fig.4D). Thus, we can complement the swarming defects of the *speA* mutant with putrescine to determine whether arginine decarboxylase contributes to alleviation of the inhibitory effects of CCCP on SWA supplemented with arginine. Importantly, arginine failed to significantly reduce the zone of CCCP inhibition of swarming for the *speA* mutant (Fig.4E). Arginine decarboxylase is therefore critical for the arginine-dependent contribution to ΔpH and *μ*H^+^ during swarming.

### Arginine decarboxylase contributes to *P. mirabilis* fitness during infection

**Figure 5:**
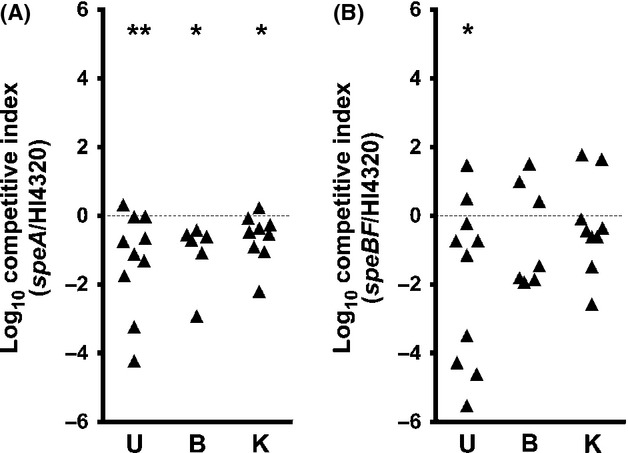
Arginine decarboxylase contributes to fitness in a mouse model of ascending UTI. CBA/J mice were infected by transurethral inoculation of a 1:1 mixture of *Proteus mirabilis* HI4320 and the *speA* mutant (A) or the *speBF* mutant (B). Urine (U), bladder (B), and kidneys (K) were collected 7 days postinoculation, and the ratio of mutant CFU mL^−1^ to wild-type CFU mL^−1^ recovered from each organ was determined and expressed as a competitive index (see Experimental Procedures). Dashed line indicates CI = 1. *P <* *0.05 and **P <* *0.01 by the Wilcoxon signed rank test.

On the basis of the contribution of arginine and *speA* to motility and prolonged survival at pH 5, we hypothesized that arginine decarboxylase may contribute to fitness in the mouse model of ascending UTI. Female CBA/J mice were infected by transurethral inoculation of 10^7^ CFU of a 1:1 mixture of *P. mirabilis* HI4320 and the *speA* mutant (Fig.5). By the Wilcoxon signed rank test, the *speA* mutant was significantly outcompeted by *P. mirabilis* Hi4320 in the urine, bladder, and kidneys 7 days postinoculation (Fig.5A). To determine whether the fitness contribution of *speA* is strictly related to a role for putrescine biosynthesis during infection, a separate group of mice were infected with a 1:1 mixture of *P. mirabilis* HI4320 and the *speBF* double mutant, which is defective in both known pathways for putrescine biosynthesis but retains functional *speA*. Notably, the *speBF* double mutant did not exhibit a severe fitness defect and was only outcompeted by the parental strain in the urine (Fig.5B). The fitness data therefore suggest that *speA* may provide a greater fitness advantage than the contribution of putrescine biosynthesis alone. We thus conclude that maintenance of ΔpH and *μ*H^+^ via arginine decarboxylase contributes to *P. mirabilis* fitness during experimental UTI.

### Proposed model of the contribution of arginine decarboxylase to *P. mirabilis* motility and fitness

**Figure 6:**
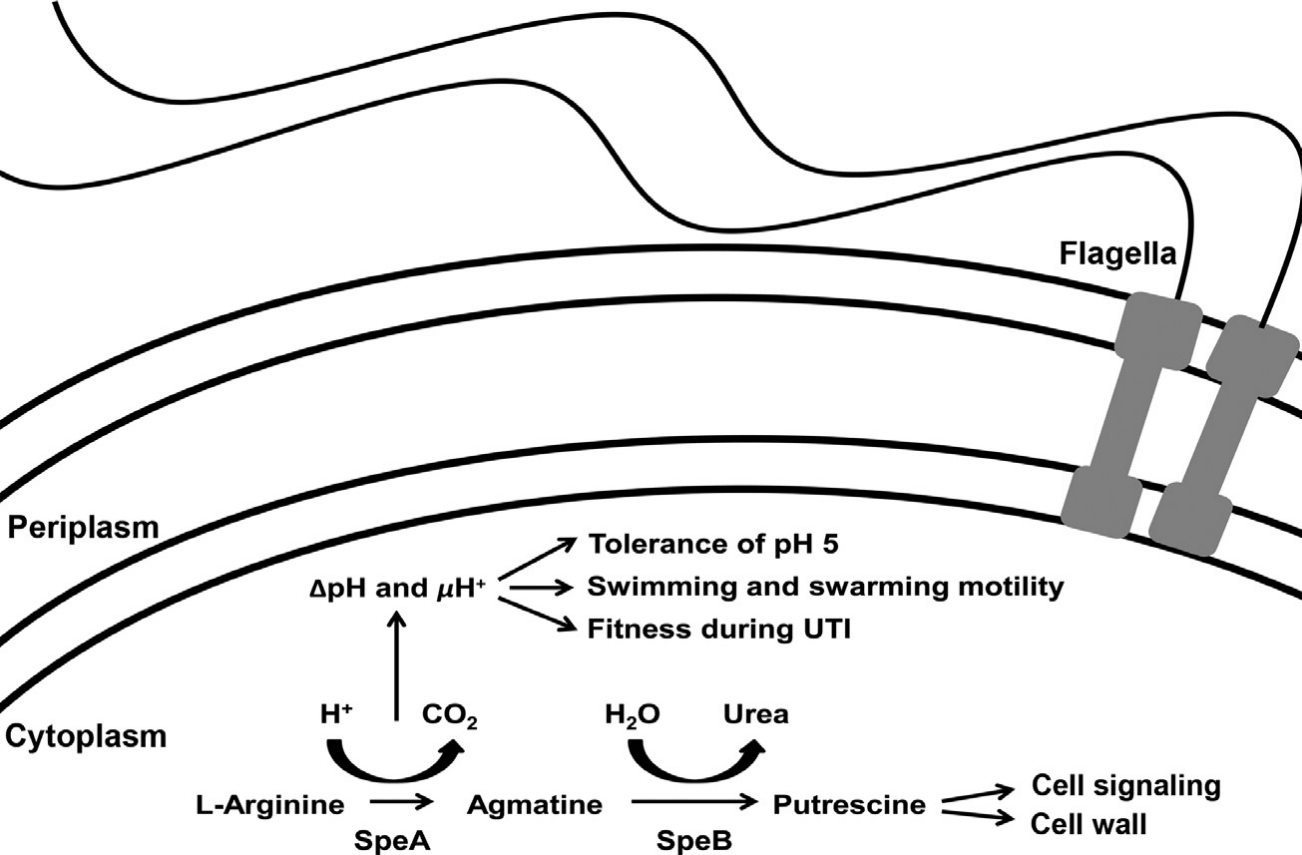
Diagram of the contribution of arginine decarboxylase to motility and fitness. Our results indicate that the arginine decarboxylase reaction has a separate contribution to motility and fitness beyond the role of this enzyme in putrescine biosynthesis and the known contributions of putrescine to swarming motility. Arginine decarboxylase (SpeA) consumes a cytoplasmic proton to produce agmatine, thereby contributing to ΔpH and *μ*H^+^. Conservation of ΔpH and *μ*H^+^ contributes to tolerance of mildly acidic conditions and provides fuel for flagellar-mediated motility, such as swimming and swarming, while also contributing to fitness in a mouse model of ascending UTI.

A graphical summary of our results is shown in Figure6. Putrescine biosynthesis is known to be important for *P. mirabilis* swarming motility as the putrescine may act as a signaling molecule for coordination of swarming (Sturgill and Rather 2004; Kurihara et al. 2013), and putrescine is also a component of the core region of *P. mirabilis* lipopolysaccharide (Vinogradov and Perry 2000) and may be important for changes in cell wall composition during swarm cell differentiation. However, our results indicate that the arginine decarboxylase reaction has a separate contribution to motility and fitness beyond the role of this enzyme in putrescine biosynthesis. Arginine decarboxylase (SpeA) consumes a cytoplasmic proton to produce agmatine, thereby contributing to ΔpH and *μ*H^+^. Conservation of ΔpH and *μ*H^+^ contributes to tolerance of mildly acidic conditions and provides fuel for flagellar-mediated motility, such as swimming and swarming. As urine is mildly acidic and flagellar-mediated motility is important for ascension from the bladder to the kidneys during UTI, both of these factors likely contribute to fitness during UTI and the observed fitness defect of the *speA* mutant.

**Figure 7:**
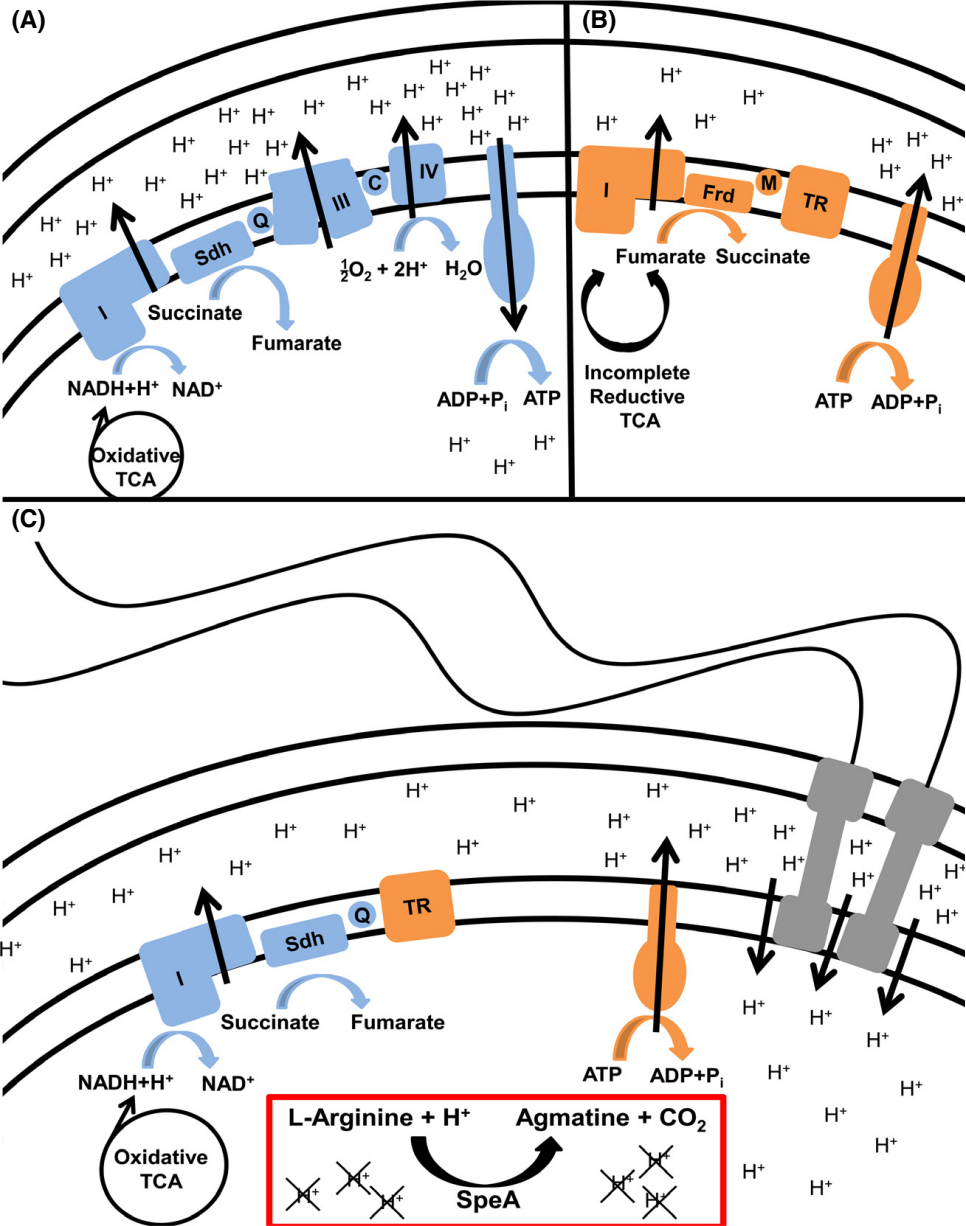
Model of *μ*H^+^ generation and maintenance during swarming. Mechanisms for generating and maintaining *μ*H^+^ are diagramed for aerobic conditions (A, blue), anaerobic conditions (B, orange), and the unusual system used during *Proteus mirabilis* swarming (C). (A) Under aerobic conditions, NADH is generated from pyruvate through a complete oxidative TCA cycle. Protons are pumped via a respiratory chain composed of NADH dehydrogenase (I), succinate dehydrogenase (Sdh), ubiquinone (Q), the cytochrome *bc* complex (III), cytochrome *c* (C), and cytochrome *c* oxidase (IV). A large proton gradient (ΔpH) is established and can be used to generate ATP. (B) When oxygen is not available, a branched TCA cycle is used and NADH will not be produced. The *P. mirabilis* anaerobic respiratory chain is likely composed of an alternative dehydrogenase (I), fumarate dehydrogenase (Frd), menaquinone (M), and a terminal reductase (TR). *μ*H^+^ can be maintained by hydrolyzing the ATP produced through fermentation to pump protons across the membrane. This process is less energetically favorable and produces a weak ΔpH and *μ*H^+^. (C) During swarming, *P. mirabilis* operates a complete oxidative TCA cycle and generates NADH, but oxygen is not used as the terminal electron acceptor. The swarming respiratory chain appears to be composed of NADH dehydrogenase (I), succinate dehydrogenase (Sdh), ubiquinone (Q), and an unknown terminal reductase (TR), possibly fumarate. ATP is most likely hydrolyzed to pump protons as the ATPase would otherwise be competing with the numerous flagella for ΔpH. While this process produces a weaker pH gradient than aerobic respiration, arginine and SpeA can be utilized as one method for consuming intracellular protons to strengthen the ΔpH and *μ*H^+^ while also producing agmatine that can be utilized for putrescine biosynthesis.

Despite being less energetically favorable than aerobic respiration, *P. mirabilis* appears to utilize an anaerobic respiratory chain during swarming (Armitage 1981; Falkinham and Hoffman 1984; Alteri et al. 2012), which would make it difficult to maintain *μ*H^+^ and drive the rotation of hundreds to thousands of flagella (Fig.7). Our results concerning the contribution of arginine decarboxylase to conservation of *μ*H^+^ indicate that *P. mirabilis* possesses additional mechanisms to fuel swarming under these unusual metabolic conditions, such as the use of l-arginine and SpeA to consume cytoplasmic protons.

## Discussion

*Proteus mirabilis* poses a significant challenge for hospitals and long-term care facilities due to its ability to quickly traverse catheters to reach the urinary tract (Stickler and Hughes 1999; Sabbuba et al. 2002; Jacobsen et al. 2008), urease-mediated alkalinization of urine leading to catheter encrustation and urolithiasis (Griffith et al. 1976; Jones et al. 1990), and persistence within the urinary tract despite catheter changes and antibiotic treatment (Warren et al. 1982; Kunin 1989; Rahav et al. 1994). This bacterium possesses numerous virulence factors that contribute to colonization and ascension of the urinary tract, including fimbriae and other adhesins, urease, hemolysin, IgA protease, siderophores and metal transport systems, and flagellum-mediated motility (Armbruster and Mobley 2012). There is also a growing appreciation for the contribution of basic metabolic processes to *P. mirabilis* virulence, including a stringent requirement for nitrogen assimilation (Pearson et al. 2011) and identification of genes involved in carbohydrate and amino acid metabolism by signature-tagged mutagenesis studies (Burall et al. 2004; Himpsl et al. 2008). While these systems are generally complex and interconnected, we have demonstrated the ability of a single amino acid to influence *P. mirabilis* motility and fitness.

In this study, we have described a role of arginine decarboxylase (SpeA) in conservation of proton motive force (*μ*H^+^) and fitness that can be at least partially separated from the critical role of putrescine during swarming. However, it is important to note that our results do not exclude a role for putrescine in coordination of swarming (Sturgill and Rather 2004; Kurihara et al. 2013). Even though SpeA was determined to be required for swimming motility and contributes to *μ*H^+^, the swarming defect of the *speA* mutant can still be complemented by exogenous agmatine or putrescine. This finding indicates that putrescine is more important in the hierarchy of swarm signals than the *μ*H^+^ contribution of SpeA on SWA. However, the contribution of SpeA to *μ*H^+^ must be substantial for the *speA* mutant to have such a severe defect in swimming motility that was not observed for the *speB* mutant. We have clearly demonstrated that *speA* contributes to arginine-dependent alleviation of the effects of CCCP during incubation at pH 5, during swimming motility, and during swarming, while *speB* does not significantly impact response to arginine under any of these conditions. The results of the animal studies also indicate that SpeA provides a greater contribution to fitness during experimental UTI than putrescine biosynthesis alone. However, our data also do not exclude a role for putrescine during infection. Wild-type *P. mirabilis* upregulates putrescine importers during experimental UTI (Pearson et al. 2011), so the *speBF* double mutant likely procures putrescine from the host if needed.

The contribution of arginine to *μ*H^+^ and motility is notable as it represents one possible explanation for the ability of *P. mirabilis* to fuel motility despite the unusual energetics of swarming. *Proteus mirabilis* swarm cells are thought to be entirely devoted to flagellar-mediated motility (Armitage 1981; Pearson et al. 2010), yet they appear to utilize energy pathways that do not require aerobic cytochromes and instead involve anaerobic electron transport chain components that would not be as energetically favorable as aerobic respiration (Alteri et al. 2012). Thus, *P. mirabilis* must have adapted alternate pathways to support the magnitude of *μ*H^+^ required to fuel the hundreds to thousands of flagellar motors on a swarm cell. We propose that arginine decarboxylation is one such pathway, contributing to ΔpH and *μ*H^+^ during swarming through the consumption of cytoplasmic protons, as shown in Figure7. It is, however, important to note that other mechanisms for conservation of *μ*H^+^ during swarming are also likely utilized by *P. mirabilis*, and there may be additional mechanisms by which arginine affects *μ*H^+^ and motility as the *speA* mutant was affected by arginine to some extent in all experiments.

There is a growing appreciation for the contribution of basic physiological processes to bacterial pathogenicity, and our findings clearly demonstrate that a single amino acid can have pleiotropic effects that contribute to fitness. For instance, the addition of 0.1 mmol/L arginine to agar plates made from pooled human urine is sufficient to promote extensive swarming (Armbruster et al. 2013), and the current study underscores the importance of arginine for both forms of flagellar-mediated motility as well as fitness during infection. The concentration of arginine in normal human urine is ∼0.01–0.2 mmol/L (Ren and Yan 2012; Liu et al. 2013), so *P. mirabilis* may use arginine decarboxylase as one mechanism to promote motility on urine-bathed catheters or within the host urinary tract. Considering the contribution of *speA* to fitness in the mouse model of ascending UTI, arginine decarboxylation may represent a new target for prevention of *P. mirabilis* swarming on catheters or possibly for therapeutic intervention during infection. In conclusion, our findings indicate that *P. mirabilis* has co-opted pathways outside of the respiratory chain to conserve the proton gradient (ΔpH) and *μ*H^+^ in order to support motility and fitness.

## Supporting Information

**Figure S1.** Arginine decarboxylase is required for optimal growth at pH 5 and response to arginine. *Proteus mirabilis* HI4320 (A) and the *speA* mutant (B) were diluted 1:100 in unbuffered LB (pH 7) versus LB adjusted to pH 5 with or without 10 mmol/L arginine. Cultures were incubated at 37°C with continuous shaking, and OD_600_ readings were taken every 15 min for 18 h. Error bars indicate mean ± SD for one representative experiment with five replicates.

**Table S1.** Primers used in this study.
