# Supplementary figures and images for: Arginine promotes Proteus mirabilis motility and fitness by contributing to conservation of the proton gradient and proton motive force
Source: Microbiologyopen. 2014 Aug 7;3(5):630–41. doi: 10.1002/mbo3.194 (PMC4234256; doi:10.1002/mbo3.194)

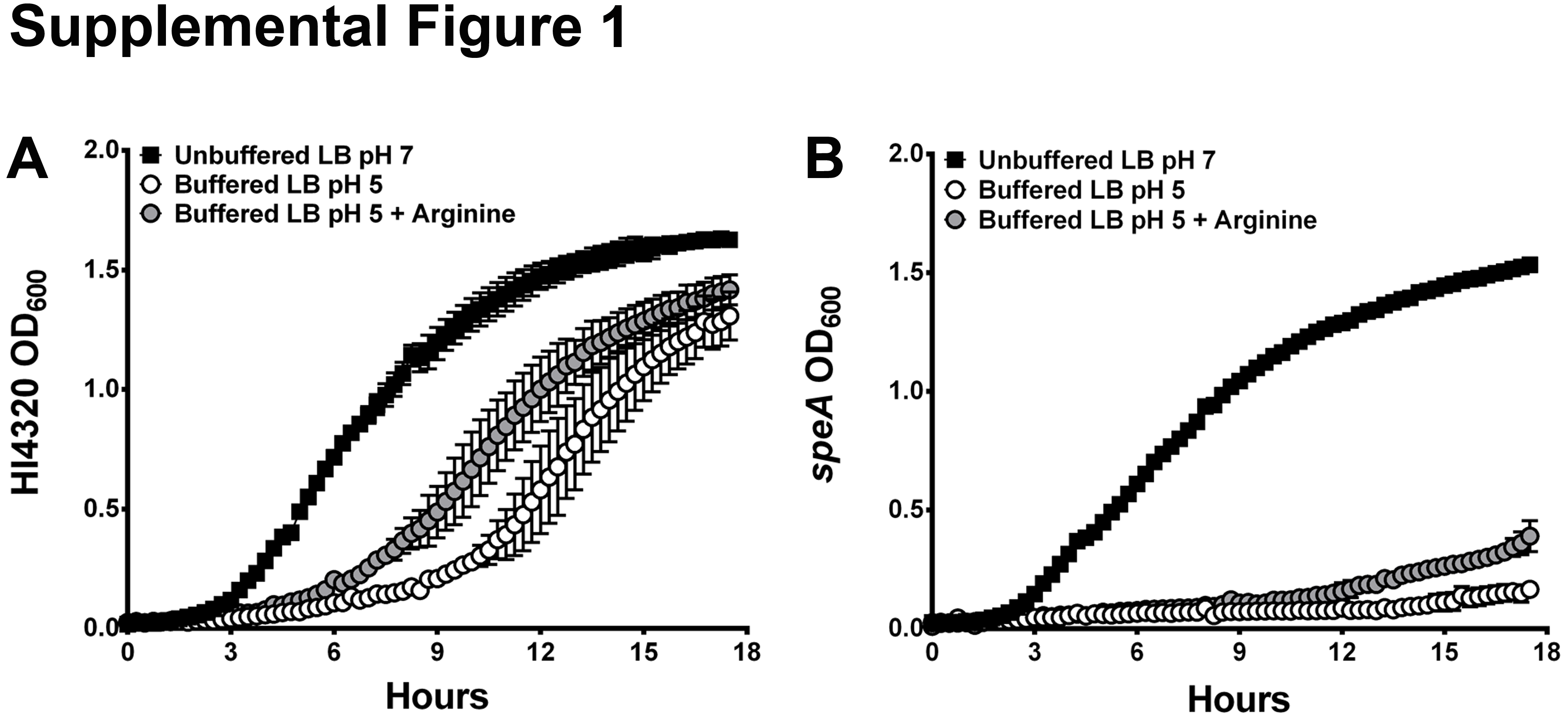

Supplement: Supplementary file 1 [file mbo30003-0630-sd1.tif]
