# Supplementary material for: Arginine promotes Proteus mirabilis motility and fitness by contributing to conservation of the proton gradient and proton motive force
Source: Microbiologyopen. 2014 Aug 7;3(5):630–41. doi: 10.1002/mbo3.194 (PMC4234256; doi:10.1002/mbo3.194)
Supplement: Supplementary file 2 [file mbo30003-0630-sd2.docx]

**Supplemental Table 1. Primers used in this study.**

| **Primer** | **Sequence** |
| --- | --- |
| *speA*-IBS | AAAAAAGCTTATAATTATCCTTACTTATCTGACATGTGCGCCCAGATAGGGTG |
| *speA*-EBS1d | CAGATTGTACAAATGTGGTGATAACAGATAAGTCTGACATACTAACTTACCTTTCTTTGT |
| *speA*-EBS2 | TGAACGCAAGTTTCTAATTTCGGTTATAAGTCGATAGAGGAAAGTGTCT |
| *ydgI*-IBS | AAAAAAGCTTATAATTATCCTTATCTTACTTAGTCGTGCGCCCAGATAGGGTG |
| *ydgI*-EBS1d | CAGATTGTACAAATGTGGTGATAACAGATAAGTCTTAGTCATTAACTTACCTTTCTTTGT |
| *ydgI*-EBS2 | TGAACGCAAGTTTCTAATTTCGGTTTAAGATCGATAGAGGAAAGTGTCT |
| *artM-*IBS | AAAAAAGCTTATAATTATCCTTAGCTTACTCAACAGTGCGCCCAGATAGGGTG |
| *artM*-EBS1d | CAGATTGTACAAATGTGGTGATAACAGATAAGTCTCAACACTTAACTTACCTTTCTTTGT |
| *artM*-EBS2 | TGAACGCAAGTTTCTAATTTCGATTTAAGCTCGATAGAGGAAAGTGTCT |
| *speA*-ver-F | GTTATTTGGGCGATTGGTGATG |
| *speA*-ver-R | CTGCTCTTGGAGTGCTTTATCT |
| *ydgI*-ver-F | CCTGATCTTGATGGCGGTATT |
| *ydgI*-ver-R | GCGATGGTCAGTAGGGTATTG |
| *artM*-ver-F | TGCGCTCTTAGTCGCCTTTACGTT |
| *artM-*ver-R | ACATTCGCTGTTTGTAGCGGTGAC |
